# Adenovirus-Mediated Expression of the p14 Fusion-Associated Small Transmembrane Protein Promotes Cancer Cell Fusion and Apoptosis *In Vitro* but Does Not Provide Therapeutic Efficacy in a Xenograft Mouse Model of Cancer

**DOI:** 10.1371/journal.pone.0151516

**Published:** 2016-03-17

**Authors:** Carmen M. Wong, Kathy L. Poulin, Grace Tong, Carin Christou, Michael A. Kennedy, Theresa Falls, John C. Bell, Robin J. Parks

**Affiliations:** 1 Regenerative Medicine Program, Ottawa Hospital Research Institute, Ottawa, ON, Canada; 2 Department of Biochemistry, Microbiology and Immunology, University of Ottawa, Ottawa, ON, Canada; 3 Centre for Neuromuscular Disease, University of Ottawa, ON, Ottawa, Canada; 4 Cancer Therapeutics Program, Ottawa Hospital Research Institute, Ottawa, ON, Canada; 5 Department of Medicine, University of Ottawa, Ottawa, ON, Canada; University of Helsinki, FINLAND

## Abstract

Adenoviruses (Ads) are used in numerous preclinical and clinical studies for delivery of anti-cancer therapeutic genes. Unfortunately, Ad has a poor ability to distribute throughout a tumor mass after intratumoral injection, and infects cells primarily within the immediate area of the injection tract. Thus, Ad-encoded transgene expression is typically limited to only a small percentage of cells within the tumor. One method to increase the proportion of the tumor impacted by Ad is through expression of fusogenic proteins. Infection of a single cell with an Ad vector encoding a fusogenic protein should lead to syncytium formation with adjacent cells, effectively spreading the effect of Ad and Ad-encoded therapeutic transgenes to a greater percentage of the tumor mass. Moreover, syncytium formation can be cytotoxic, suggesting that such proteins may be effective sole therapeutics. We show that an early region 1 (E1)-deleted Ad expressing reptilian reovirus p14 fusion-associated small transmembrane (FAST) protein caused extensive cell fusion in the replication-permissive 293 cell line and at high multiplicity of infection in non-permissive human lung adenocarcinoma A549 cells *in vitro*. FAST protein expression in the A549 cancer cell line led to a loss of cellular metabolic activity and membrane integrity, which correlated with induction of apoptosis. However, in an A549 xenograft CD-1 nude mouse cancer model, Ad-mediated FAST gene delivery did not induce detectable cell fusion, reduce tumor burden nor enhance mouse survival compared to controls. Taken together, our results show that, although AdFAST can enhance cancer cell killing *in vitro*, it is not effective as a sole therapeutic in the A549 tumor model *in vivo*.

## Introduction

Currently, approximately 50% of all cancer patients will succumb to the disease, clearly illustrating the need for new therapeutics. This need is perhaps best exemplified by the fact that approximately two-thirds of all human gene therapy clinical trials are directed towards cancer [1]. One of the most commonly used platforms for gene delivery is adenovirus (Ad). Unfortunately, when injected into a tumor, Ad does not travel far from the injection tract, rarely infecting cells more than 5 mm from the injection site [2]. As for many vector systems used in cancer therapy, this is a common issue [3]. One approach to improve distribution of viral vectors used in cancer therapeutics is through co-expression of fusogenic proteins. Infection of a single cell with a virus encoding a fusogenic protein can cause extensive multinucleated syncytial formation, effectively increasing the area and number of cells transduced by the virus [4]. Mixing of cytoplasmic contents of fusing cells means that any gene product encoded within the vector is efficiently transferred to all cells fused into the syncytium. Moreover, fusogenic proteins can act as effective single-agent therapeutics, as the process of fusion ultimately compromises cell function and induces cell death [4,5]. In the context of oncolytic virus (OV), in addition to normal tumor cell lysis resulting in release of virus, an infected cell can “spread” its infectious payload through direct cell-cell fusion. In studies using such diverse OV as vesicular stomatitis virus (VSV), herpes simplex virus, and vaccinia virus [6,7,8], and fusogenic proteins from human immunodeficiency virus, Newcastle disease virus and gibbon-ape leukemia virus (GALV) [6,9,10], improved efficacy has been achieved.

Several groups have attempted to enhance Ad spread in tumors through expression of cell fusion proteins. Expression of the GALV fusogenic envelope glycoprotein from a replication competent Ad5 based vector promoted cell-cell fusion, enhanced cancer cell killing *in vitro* and reduced tumor burden in mice harbouring tumor xenografts, relative to the control virus [9]. Expression of the respiratory syncytial virus (RSV) fusion protein from a replication defective Ad vector reduced tumor burden in a mouse model of colorectal cancer [5], suggesting that fusogenic proteins have the added benefit of being effective sole anti-cancer molecules. However, a limitation of this approach is that these fusogenic proteins are relatively large (~2 kb) and may not be easily accommodated in E1-deleted Ad vectors when paired with large upstream regulatory regions necessary to promote tumor-specific expression or multimodal treatments utilizing additional therapeutic genes delivered in the same vector. Ad have a limited cloning capacity; E1-deleted vectors can accommodate at most ~8 kb of foreign DNA [11,12]. As such, smaller proteins that have the ability to cause cell fusion may be more ideal.

A candidate fusogenic protein to enhance the efficacy of Ad for cancer is the p14 fusion-associated small transmembrane (FAST) protein. The p14 FAST protein is a 125 amino acid (375 bp), nonstructural protein from reptilian reovirus that can mediate cell-cell membrane fusion [13]. This fusogenic protein is a type III single pass transmembrane protein with a hydrophobic myristylated N terminus, and a C-terminal domain composed of a highly basic, membrane-proximal region and a C-terminal proline-rich region. Expression of p14 FAST protein in cells results in extensive cell fusion, and induces apoptosis-dependent membrane permeability [13,14]. The FAST protein has already demonstrated an ability to enhance the efficacy of other vector systems for cancer. A VSV encoding p14 FAST protein showed increased replication and neuropathogenesis *in vivo* compared to the control virus, indicating the FAST protein can act as a virulence factor to promote virus spread [15]. Enhanced efficacy was observed on coinfection of an oncolytic VSVΔ51 [16] expressing p14 FAST protein and a doubly-deleted vaccinia virus (VV) (deficient in the viral thymidine kinase and vaccinia growth factor [17]) [8]. In 786-O kidney cancer cells, coinfection of these two viruses increased the yield of VV titre by ~100 fold relative to the combination of VV and native VSVΔ51, and also enhanced cell killing in primary human colon tumor cells [8]. The small size of the FAST protein and its demonstrated ability to enhance virus spread and efficacy make it an ideal candidate for helping improve the spread of Ad-encoded gene products through a tumor. In this study, we explore the ability of Ad-mediated expression of p14 FAST protein to promote cell fusion in tissue culture and as a sole therapeutic in a murine xenograft model of cancer.

## Materials and Methods

### Cell culture

The 293 [18], 293N3S [19] and A549 human lung adenocarcinoma cells were grown in Minimum Essential Medium (MEM) (Sigma Aldrich) containing 10% (v/v) Fetal Bovine Serum (FBS) (Sigma Aldrich), 2mM GlutaMAX (Invitrogen) and 1x antibiotic-antimycotic (Invitrogen). Ad infected cells were maintained in MEM supplemented with 5% FBS, 2mM GlutaMAX and 1x antibiotic-antimycotic.

### Adenoviral constructs and adenovirus purification

**Fig 1:**
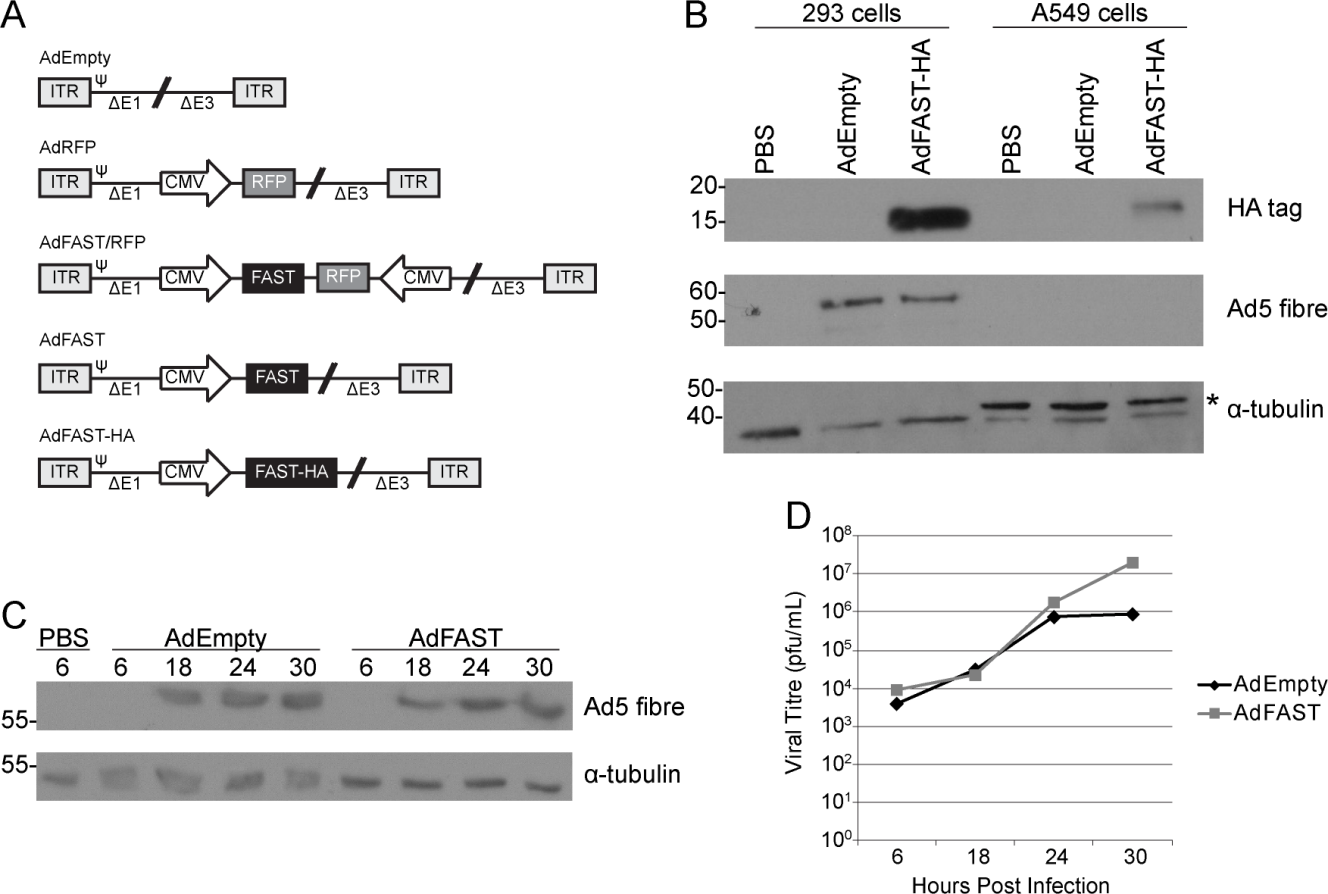
Ad-mediated FAST protein expression from an E1-deleted vector does not affect virus growth or yield. **A)** All viruses are early region 1 (E1) and early region 3 (E3) deleted. AdFAST-HA has a single HA tag linked to the C terminus through a glycine linker. ITR denotes inverted terminal repeats and Ψ is the packaging signal. RFP is the red fluorescent protein and CMV represents the cytomegalovirus enhancer/promoter. **B)** 293 and A549 cells were infected with the control virus AdEmpty or AdFAST-HA at an MOI of 10. Whole cell lysates were collected 48 hpi. Samples were probed for the HA tag, Ad5 fibre and alpha-tubulin for a loading control. The top band denoted by * in the A549 samples is non-specific. **C)** 293 cells were infected with AdEmpty or AdFAST at an MOI of 1 and whole cell lysates were collected at 6, 18, 24 and 30 hpi. Samples were probed for Ad5 fibre and alpha-tubulin was used as a loading control. **D)** Supernatants collected from 293 cells infected with AdEmpty or AdFAST at an MOI of 1 were used to conduct plaque forming assays to determine the number of viral progeny at various times post-infection.

Serotype 5 Ad constructs used for these experiments are depicted in Fig 1A. All Ad are early-region 1 (E1) and E3 deleted, and were generated using a combination of conventional and RecA-mediated bacterial cloning [20]. To summarize, the vector lacking a transgene and deleted of E1 and E3 regions is denoted as AdEmpty. The AdRFP construct encodes a monomeric Red Fluorescent Protein (mRFP), a kind gift of R. Tsien (University of California San Diego [21]), under control by the cytomegalovirus (CMV) enhancer/promoter and bovine growth hormone polyadenylation sequence (BGHpA) replacing the E1 deletion [22,23]. The FAST cDNA was removed from plasmid pVSV-FAST [8] by digestion with NcoI/NheI, and cloned into NcoI/SpeI digested pPOLY [24], generating pRP2868. pRP2868 was digested with EagI, and the fragment was cloned into NotI digested pRP2645, an Ad left end shuttle plasmid containing the CMV enhancer/promoter, a multiple cloning site and BGHpA, generating pRP2870. pRP2870 was recombined into an Ad genomic plasmid, pRP2014 [25], generating pRP2872 and the resulting virus AdFAST. A XhoI/SalI fragment from pRP2483 [22] containing a CMV-mRFP-BGHpA expression cassette was cloned into SalI digested pRP2870, with transcription directed opposite to the FAST expression cassette, generating pRP2873. pRP2873 was recombined into the pRP2014 Ad genomic plasmid, generating pRP2874 and the resulting virus AdFAST/RFP. AdFAST-HA is similar to AdFAST, but contains a C-terminal HA epitope tag. The HA tag was added with a glycine linker and amplified from pRP2868 using the sense primer 5’-CCGCCATGGGGAGTGGACC-3’ and antisense primer 5’-CTAACTAGTCTAAGCGTAGTCTGGGACGTCGTATGGGTACGAGCCACCGCCACCAATGGCTGAGACATTATCGATGTTGACG-3’. The resulting PCR fragment was digested with NcoI/SpeI and cloned into pRP2868, replacing the native FAST cDNA, generating pCW100. pCW100 was sequenced to verify its integrity. An EagI fragment from pCW100 was cloned into NotI digested pRP2645, generating pCW101, and subsequently recombined into the pRP2014 Ad genomic plasmid, generating pCW103 and the resulting virus AdFAST-HA. Vectors were propagated and purified using standard techniques [26].

### Analysis of FAST protein expression

293 and A549 cells were seeded at 1x10^6^ and 0.8x10^6^ cells per 35mm dish, respectively. Next day, the cells were infected for 1 h at a multiplicity of infection (MOI) of 10 with AdEmpty or AdFAST-HA. Following a 48 h incubation, whole cell lysates were collected using the cell lysis buffer as described by Lieber *et al*. [27]. Samples were separated by 15% SDS-PAGE and transferred onto a polyvinylidene fluoride (PVDF) membrane (Millipore). The resulting membrane was probed with a mouse anti-HA tag monoclonal antibody (1:1000, Cell Signaling #2367) and goat anti-mouse antibody conjugated to horseradish peroxidase (HRP) (1:10 000, Bio-Rad #170–6516). To confirm Ad replication, the membrane was reprobed for Ad5 fibre (1:20 000 mouse anti-fibre monoclonal antibody, clone 4D2, Neomarkers). The membrane was also probed with antibody to α-tubulin to confirm equal loading (1:5000 rabbit anti-α-tubulin antibody, AbCam #ab15246, 1:5000 goat anti-rabbit IgG conjugated to HRP, Bio-Rad #170–6515). Blots were developed using the Pierce Enhanced Chemilumescent (ECL) Western Blotting Substrate (Thermo Scientific). Densitometry was performed using AlphaEaseFC (Alpha Innotech).

### Adenoviral gene expression and viral progeny

293 cells were plated at a density of 1x10^6^ cells per 35mm dish. Cells were infected at an MOI of 1 with AdEmpty or AdFAST the next day for 1 h. Cells were washed two times with PBS before adding 5% FBS/MEM. Whole cell lysates were collected at varying time points using cell lysis buffer as described above and probed for Ad5 fibre to compare adenoviral gene expression between the two vectors. Supernatants were also collected to determine viral progeny titre using plaque assays [26].

### Giemsa staining

293 and A549 cells were seeded in 35mm dishes at densities of 1x10^6^ and 0.8x10^6^ cells per dish, respectively, and grown to confluency. 293 cells were infected at an MOI of 1 with AdEmpty or AdFAST and stained with Giemsa stain (Merck Millipore) 18 hpi. A549 cells were infected at an MOI of 50 with AdEmpty or AdFAST and subjected to Giemsa staining 48 hpi. Stained cells were visualized using the 20x objective of the Zeiss Axiovert 200M microscope. The fusion index was calculated using the formula: (N-S)/T x 100%, where N is the number of nuclei in syncytium, S is the number of syncytia and T is the total number of nuclei [28].

### Fluorescence microscopy and quantification

Thirty-five mm dishes of 293 and A549 cells were seeded at a density of 0.6x10^6^ cells. Next day, the cells were infected with AdRFP or AdFAST/RFP at varying MOI. The cells were visualized at varying times post infection using a Zeiss Axiovert 200M microscope. Images were taken using a 20x objective and compiled in Adobe Photoshop CS4. For the A549 co-culture assay, A549 cells were infected with AdRFP or AdFAST/RFP at an MOI of 50. After 6 hours, the infected A549 cells were mixed at a 1:1 ratio with A549::WPI cells, which constitutively express the green fluorescent protein (GFP) from an integrated lentivirus vector. Cocultures were observed using fluorescence microscopy at 24 h intervals. Images were captured and processed as described above. Image J [29] was used to calculate the area fluorescing green for coculture experiments.

### MTS metabolic activity assays

293 and A549 cells were seeded at 2.5x10^4^ and 2x10^4^ cells per well, respectively, in 96 well plates. Next day, the cells were infected at varying MOI with AdEmpty or AdFAST in 25 μL inoculum for 1 h, followed by addition of MEM supplemented with 5% FBS to a final volume of 100 μL. Metabolic activity was assessed using the CellTiter 96® AQueous Non-Radioactive Cell Proliferation Assay (Promega) at 72 hpi using a 1 h incubation with MTS substrate (3-(4,5-dimethylthiazol-2-yl)-5-(3-carboxymethoxyphenyl)-2-(4-sulfophenyl)-2H-tetrazolium). A second set of cells were infected at an MOI of 10 with AdEmpty or AdFAST and the relative metabolic activity was monitored using the MTS assay over 96 h at 24 h intervals. Absorbance was determined at 490 nm using the SpectraMax 190 plate spectrophotometer (Molecular Devices).

### Assay of membrane integrity

A549 cells were seeded at a density of 2x10^4^ cells per well in 96 well plates and grown to confluency. Medium was removed and cells were infected with AdEmpty or AdFAST at a MOI of 1, 5, 10, 50 or 100 in 25 μL inoculum for 1 h. The VSVΔ51 virus [16] was used as a positive control. Medium was added to a final volume of 125 μL and the cells were incubated at 37°C in 5% CO_2_ for 72 h. Prior to collecting the supernatants, a series of wells containing uninfected cells were lysed with 9% Triton X-100 to provide a maximum release control. All treatments were completed in triplicate. Fifty μL of supernatant was transferred to a new 96 well plate and the CytoTOX-ONE™ Homogeneous Membrane Integrity Assay kit (Promega) was used to assess membrane integrity. Fluorescence was determined using the 544 nm excitation filter and 590 nm emission filter with a FluoSTAR Galaxy fluorimeter (BMG Labtech).

### Analysis of cleaved caspase-3 protein expression

A549 cells were seeded at 0.8x10^6^ cells per 35mm dish and grown to confluency. Cells were infected for 1 h with AdEmpty or AdFAST at an MOI of 10. As positive control, cells were treated with 100 μM etoposide for 24 hr or 1 μM staurosporine for 6 hr. Whole cell lysates were collected at varying time post-infection with 2x Laemmli loading buffer (62.5mM Tris HCl pH 6.8, 25% glycerol, 2% SDS, 0.01% bromophenol blue, 5% β-mercaptoethanol). Samples were resolved by 15% SDS-PAGE and transferred onto a PVDF membrane (Millipore). Membranes were probed with rabbit anti-cleaved caspase-3 monoclonal antibody (1:1000, Cell Signaling #9664) or full-length caspase 3 (1:1000, Cell Signaling #9662) and 1:5000 goat anti-rabbit IgG conjugated to HRP (Bio-Rad #170–6515). Alpha-tubulin was used as a loading control (1:5000, CalBiochem #CP06, 1:10 000 goat anti-mouse IgG conjugated to HRP, Bio-Rad #170–6516). Blots were developed and densitometry was conducted as described above.

### Nude mouse studies

Six week old CD-1 nude mice were obtained from Charles River Laboratories (Wilmington, MA), and allowed to acclimate on site for at least one week prior to initiation of animal procedures. Mice were anesthetized (isoflurane) prior to injection of tumor cells and virus. Mice were injected in the right hind flank with 10^6^ A549 cells resuspended in 100 μL PBS. When tumors reached approximately 5x5mm in size, mice were injected intratumorally with PBS, or 5x10^8^ plaque forming units (pfu) of AdEmpty or AdFAST in 50 μL. Tumor size was monitored three times a week using digital calipers. Endpoint was considered when the tumor reached 1687 mm^3^ by volume (length x width^2^ /2) or ulceration was observed. This endpoint was applied to all experiments reported in the manuscript, including the survival analysis. A second group of mice were injected when A549 tumors reached 7x7 to 10x10 mm in size with the same virus dose as above. The second set of mice was sacrificed 5 days post injection for histological analysis of tumor and liver samples. Samples were fixed with 10% formalin overnight and submerged in 70% ethanol until processed. Tissues were processed at the Morphology Unit of the Department of Pathology and Laboratory Medicine at University of Ottawa.

Experimental animals were cared for in the Animal Care Facility at the University of Ottawa. Mice were kept in a conventional animal house in groups of 2–4, with a constant room temperature of 24°C and a 12 h dark/light cycles, and with free access to food and water. Mice were monitored daily by animal facility professionals, and additionally monitored by the researchers once every 3 days for tumor measurement, and daily when tumor size was nearing endpoint (1687 mm^3^ by volume (length x width^2^ /2)). Animal facility reported to researchers any identified animal recommended for sacrifice due to advancement of tumor burden or generalized distress. Mice were euthanized or sacrificed using carbon dioxide, followed by cervical dislocation.

### Ethics Statement

The animal protocol (ME-2258) was approved by the University of Ottawa Animal Care Committee and conducted according to the guidelines of the Canadian Council on Animal Care’s Guide to the Care and Use of Experimental Animals, and the Animals for Research Act.

### Statistical analysis

SigmaStat 2.0 was used to determine statistical significance. One-way ANOVA tests and Tukey’s test were applied when normality was observed while Kruskal-Wallis one-way ANOVA on Ranks was used when samples did not follow a normal distribution. Dunn’s test was used in place of Tukey’s test when required. The student’s t-test was used where applicable. Statistical analyses for the animal studies were conducted using Graphpad Prism 6.0. The log-rank test was used to determine statistical significance of the Kaplan-Meier survival curve. All tests were conducted at p = 0.05.

## Results

### Ad-mediated FAST expression in mammalian cells causes extensive cell fusion under replicative conditions

The viral constructs used in this study are shown in Fig 1A. All viruses used in this study are deleted of early region 1 (E1) and E3. Such viruses can replicate in the E1-complementing 293 cell line but not in most other cells, including A549 cells. To confirm FAST protein was expressed from our viral constructs, we infected 293 and A549 cells with AdEmpty and AdFAST-HA at an MOI of 10 and examined the relative FAST protein expression 48 hours later by immunoblot of whole cell protein lysates (Fig 1B). As expected, AdFAST-HA produced a protein of appropriate size in both 293 and A549 cells, although the level of FAST protein expression was higher in 293 cells. This is likely due to the increase in template copy number caused by active virus replication in 293 cells.

To confirm that FAST protein expression does not affect Ad replication, we performed a time course of infection and monitored the expression of fibre, a late gene that is expressed only after active DNA replication, by immunoblot (Fig 1C) and virus yield by plaque assay (Fig 1D). No difference in fibre expression was observed between AdEmpty and AdFAST infected cells at late time points. In addition, there was no difference in the number of Ad progeny up to 24 hpi as determined using a plaque forming assay (Fig 1D). Thus, FAST protein expression did not affect virus replication and progeny production for an E1-deleted Ad vector in 293 cells.

**Fig 2:**
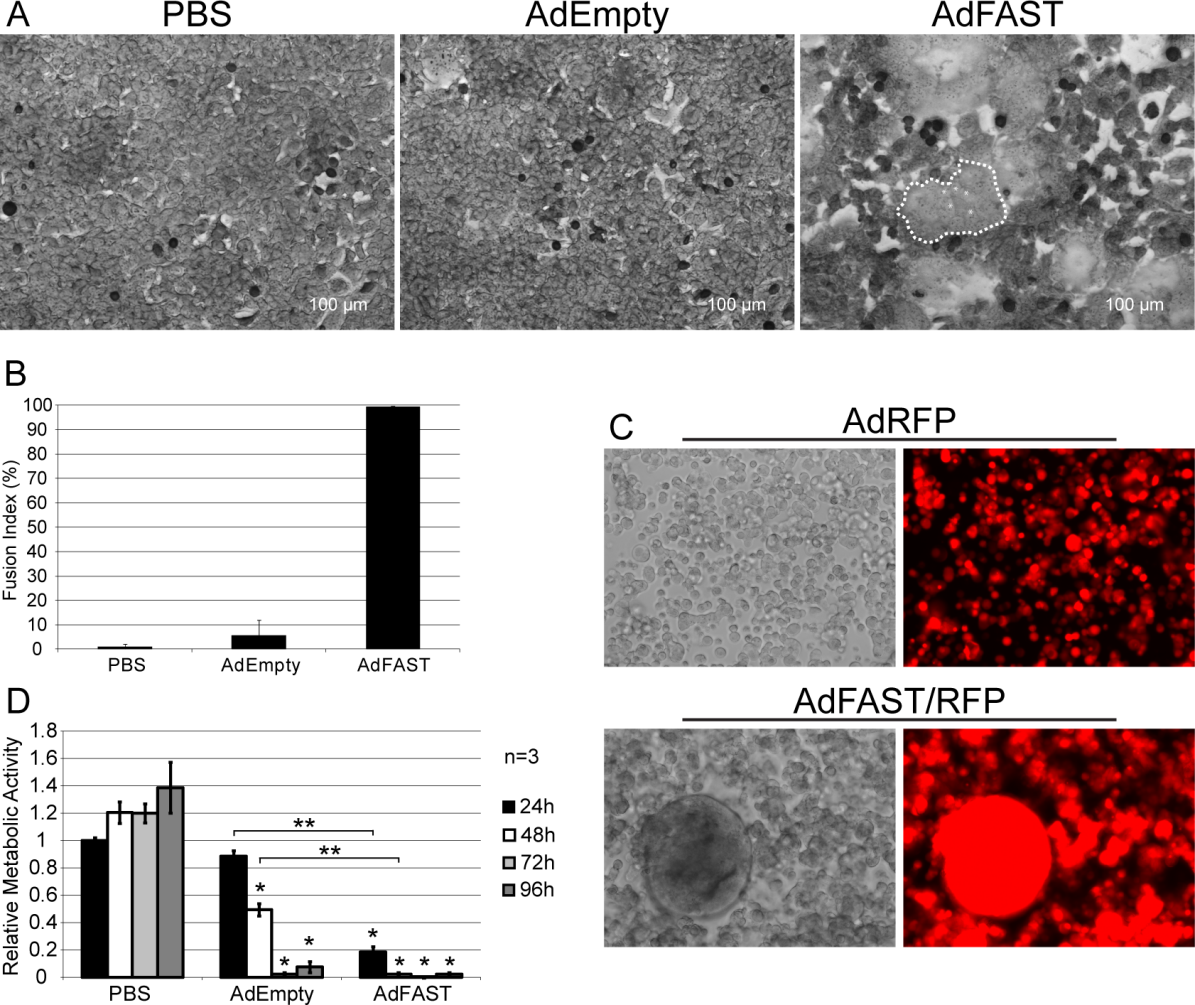
FAST protein expression induces extensive cell fusion in 293 cells. **A)** 293 cells were infected with AdEmpty or AdFAST at an MOI of 1 and stained with Giemsa stain 18 h later. Images were captured using bright field microscopy (20x objective). A region of fused cells for the AdFAST-treated cells is outlined with a dotted line, and several nuclei within the syncytium are indicated with asterisks (*) **B)** Fusion index of 293 cells infected with AdEmpty or AdFAST. The fusion index for two fields of view were determined, and the average with the standard deviation is depicted in the graph. **C)** 293 cells were infected with AdRFP or AdFAST/RFP at MOI 10 and observed using fluorescence microscopy at 48 h post infection. All images were taken using a 20x objective. **D)** 293 cells were infected at a MOI of 10 with AdEmpty or AdFAST and relative metabolic activity was determined using an MTS assay over a 96 h interval every 24 h. Experiments were completed in triplicate and the average of three independent experiments is shown (n = 3). Values were normalized to mock infected cells at 24 hpi. Error bars denote the standard error of the mean. *p<0.05 compared to mock infected cells. **p<0.05 when comparing AdFAST to AdEmpty infected cells.

We next examined whether expression of FAST protein from an Ad vector can promote cell fusion, initially using 293 cells. Both AdEmpty and AdFAST can replicate in this cell line, which provides very high levels of FAST protein expression (Fig 1B). We infected 293 cells with AdEmpty or AdFAST at an MOI of 1 and stained the cells with Giemsa stain 18 hpi (Fig 2A). At this time point, cells infected with AdEmpty showed no discernable difference relative to PBS-treated control cells. In contrast, cells treated with AdFAST showed evidence of cell fusion, and the formation of multinucleated syncytia (fusion index of 99.2%) at this early time point (Fig 2B). With a longer incubation period, the fused cells from the AdFAST/RFP treatment lifted from the plate and adopted a large spherical structure, which was not observed for cells treated with control virus (Fig 2C). Mock infected cells did not show any signs of fusion or fluorescence (not shown). Live imaging analysis of AdRFP- and AdFAST/RFP-infected cells also confirmed AdFAST/RFP-mediated fusion and enhanced cytopathic effect (S1 Movie and S2 Movie). Thus, expression of the FAST protein from an Ad vector in 293 cells caused extensive cell fusion.

We next examined the effect of Ad-mediated FAST protein expression on cell metabolic function. 293 cells were infected at an MOI of 10 with AdEmpty or AdFAST (or treated with PBS), and metabolic activity was examined at 24 h intervals using an MTS assay. As shown in Fig 2D, although treatment with both vectors caused a decrease in metabolic activity relative to PBS-treated cells, AdFAST-treated cells showed a dramatic decrease in metabolic activity compared to AdEmpty at both 24 and 48 hpi. At later time points (72–96 hpi), both viruses showed similar decrements in metabolic activity, likely due to the effects of progressive virus replication in the 293 cell line. Taken together, these data indicate that high-level expression of FAST protein from an Ad vector during replication permissive conditions in the 293 cell line leads to extensive cell fusion and decreased cellular metabolic activity.

### Ad-mediated FAST protein expression in A549 cells induces cell-cell fusion

**Fig 3:**
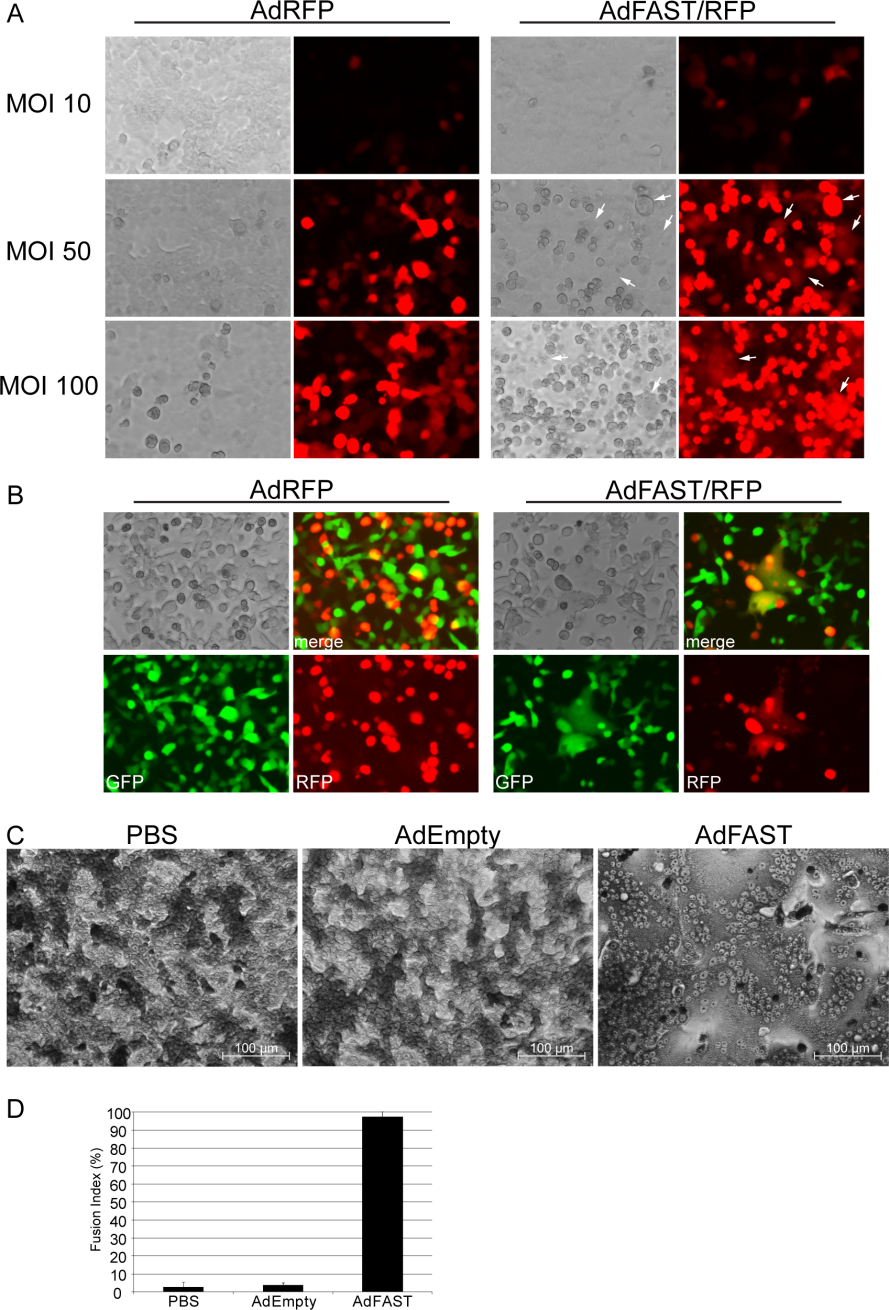
Ad-mediated FAST protein expression induces cell fusion in A549 cells. **A)** A549 cells were infected with AdRFP or AdFAST/RFP at an MOI of 10, 50 or 100. Fluorescence microscopy was used to visualize cells 48 hpi (20x objective). Fused cells are indicated by the white arrows. **B)** A549 cells infected with AdRFP or AdFAST/RFP at an MOI of 10 were cocultured 6 hpi at a 1:1 ratio with A549 cells constitutively expressing GFP. After 72 h of coculturing, cells were visualized using fluorescence microscopy with a 20x objective. The upper right image shows the merged image while the two bottom panels show GFP and RFP fluorescence. **C)** A549 cells were infected with AdEmpty or AdFAST at an MOI of 50 and stained with Giemsa stain after 48 hpi. Cells were visualized using bright field microscopy with a 20x objective. Two independent experiments were conducted with representative images depicted. **D)** The fusion index of Giemsa-stained A549 cells infected with AdEmpty or AdFAST at an MOI of 50 was calculated from two fields of view from each independent experiment (n = 2). The average fusion index is shown with the standard deviation.

Infection of 293 cells with AdFAST led to high levels of protein expression (Fig 1B), resulting in extensive cell-cell fusion (Fig 2A–2C) and decreased cellular metabolic activity (Fig 2D) relative to cells treated with AdEmpty. Under non-replicating conditions in A549 cells, Ad-mediated FAST protein expression was noticeably reduced (Fig 1B). We therefore examined whether AdFAST could also mediate cell fusion in this cell line. We used three assays to assess cell fusion. First, A549 cells were infected with AdRFP or AdFAST/RFP at varying MOI and the cells were simply visualized at 48 hpi using fluorescence microscopy. Only isolated regions of the AdFAST/RFP-treated monolayer showed large cells indicative of cell fusion (Fig 3A, white arrows). To confirm that cell-cell fusion was occurring in A549 cells, we next performed a cell coculture assay. AdFAST/RFP- or AdRFP-infected A549 cells were cocultured at a 1:1 ratio with A549 cells that constitutively expressed GFP (A549::WPI) (Fig 3B). Incubation of AdRFP-infected cells with A549::WPI cells showed some evidence of merged fluorescence signal (yellow), but this appeared to be exclusively due to cell overlap. In contrast, coculture with AdFAST/RFP-infected cells resulted in large areas of yellow signal, suggesting true fusion into multinucleated syncytium.

Finally, A549 cells were treated at an MOI of 50 with AdEmpty or AdFAST and, 48 h later, the cells were stained with Giemsa (Fig 3C). Once again, cells infected with AdEmpty showed no discernable difference relative to PBS-treated control. Quantitatively, the fusion index was similar between the PBS and AdEmpty-treated cells (i.e. 2.7% vs 3.7%, respectively) (Fig 3D). In contrast, cells treated with AdFAST showed evidence of cell fusion, and formation of multinucleated syncytia with a fusion index of 97.5%. Although a similar fusion index was observed for 293 and A549 cells, the latter required a high MOI (1 versus 50, respectively), and a longer period of incubation with virus (18 versus 72 hpi, respectively), likely due to the differences in FAST protein expression levels for replicating versus non-replicating virus, respectively. Taken together, these data indicate that Ad-mediated expression of FAST protein in A549 cells does result in cell-cell fusion.

### Treatment of A549 cells with AdFAST decreases cellular metabolic activity and membrane integrity, and induces apoptosis

**Fig 4:**
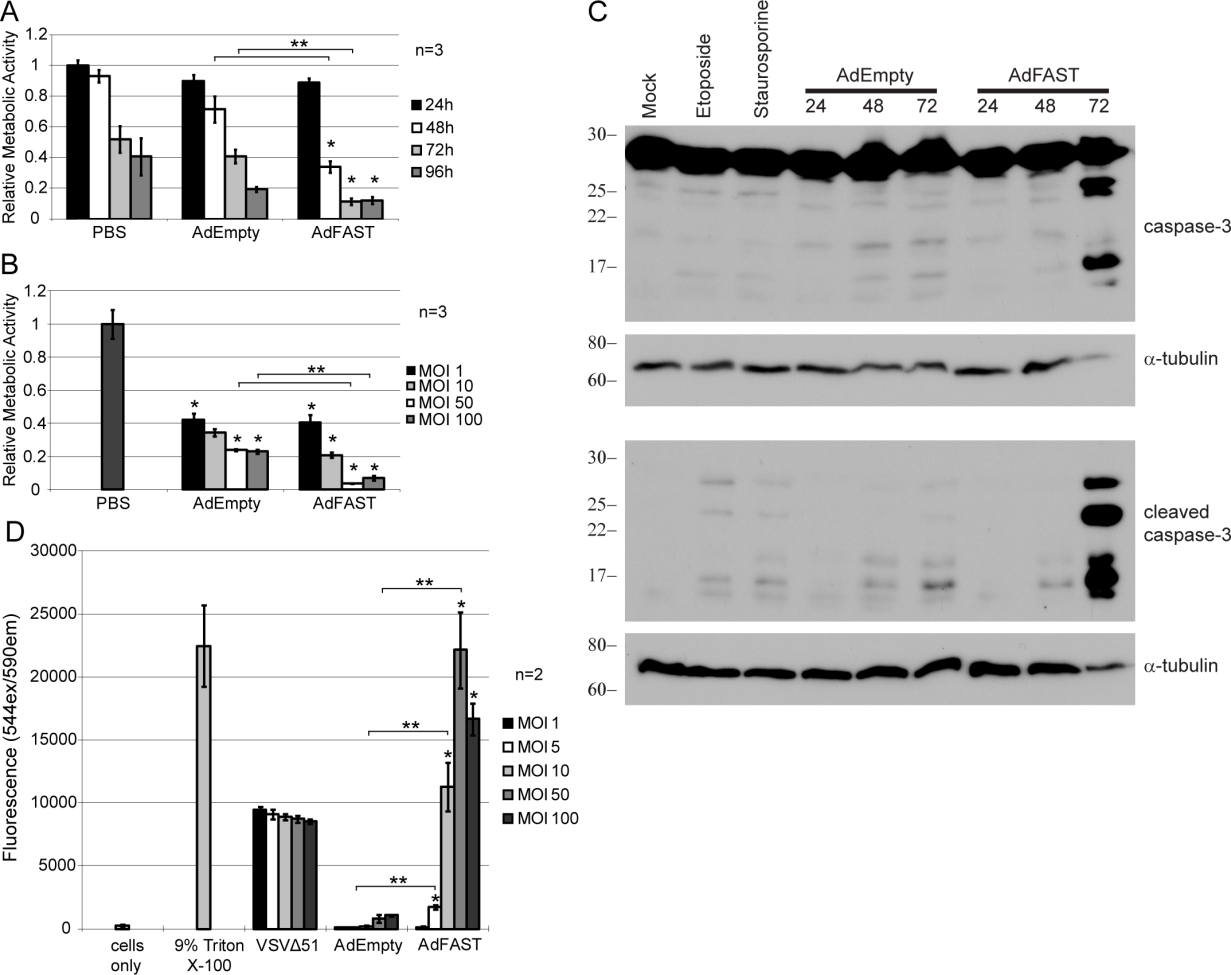
FAST protein expression in A549 cells decreases cellular metabolic activity, promotes apoptosis and concomitant loss of membrane integrity. **A)** A549 cells were infected at an MOI of 10 with AdEmpty or AdFAST. Cellular metabolic activity was assessed every 24 h until 96 hpi using an MTS assay. The average of three independent experiments (n = 3) done in triplicate is plotted with the standard error of the mean. Values were normalized to mock infected cells at 24 hpi. *p<0.05 compared to mock infected cells at their corresponding timepoints. **p<0.05 comparing AdFAST to AdEmpty infected cells. **B)** A549 cells were infected with AdEmpty or AdFAST at an MOI of 1, 10, 50 or 100. An MTS assay was conducted 72 hpi. The average of three independent experiments (n = 3) done in triplicate ± the standard error of the mean is shown with normalization to mock infected cells. *p<0.05 compared to mock infected cells. **p<0.05 comparing AdFAST to AdEmpty treated cells. **C)** A549 cells were treated with AdEmpty or AdFAST at an MOI of 10, or left uninfected, and crude protein extracts prepared 24 hr later and examined for total or cleaved caspase 3 by immunoblot. As positive controls, A549 cells were treated with 100 μM etoposide for 24 h, or 1 μM staurosporine for 6 h. Alpha-tubulin served as a loading control. **D)** A549 cells were infected with AdEmpty, AdFAST or VSVΔ51 using a range of MOI. Relative cell membrane integrity was measured based on the lactate dehydrogenase levels in the supernatant 72 hpi. The average of two independent experiments (n = 2) is shown with each experiment done in triplicate. Error bars denote the standard error of the mean. *p<0.05 compared to cells only. **p<0.05 when comparing AdFAST to AdEmpty infected cells.

In 293 cells under conditions in which FAST protein is expressed at very high levels, we observed a significant decrease in cellular metabolic activity for AdFAST- relative to AdEmpty-treated cells (Fig 2D). We next asked whether a similar effect was observed in A549 cells in which FAST protein is expressed at lower levels due to the inability of the virus to replicate in this cell line (Fig 1B). A549 cells were infected with AdEmpty or AdFAST at an MOI of 10 and metabolic activity was assessed by MTS assay every 24 h. For PBS-treated cells, we observed a decrease in metabolic activity over the 4-day course of the assay, likely reflecting reduced metabolic activity as the cells reached confluency and underwent contact inhibition (Fig 4A). For cells treated with AdEmpty, no difference in metabolic activity, relative to PBS-treated control cells, was observed. In contrast, AdFAST-treated cells had significantly decreased metabolic activity compared to PBS-treated cells at all time points beyond 24 hr, and reduced activity compared to AdEmpty-infected cells at 48 hpi and 72 hpi (p<0.05) (Fig 4A). These data suggest that under conditions in which FAST protein is expressed at lower levels, time may be required for the protein to accumulate to sufficient levels to mediate fusion and impact metabolic function within the cell. Consistent with this, we observed that treating cells with a higher MOI of AdFAST, which would lead to higher cellular levels of FAST protein, had a greater negative effect on metabolic activity (p<0.05) relative to AdEmpty (Fig 4B). We also examined the metabolic effects of Ad-mediated FAST protein expression in four other human cell lines—kidney adenocarcinoma 786-O, glioblastoma SNB75, and two normal lung cell lines (U87N and U116N [30]) (S1 Fig). For the two human cancer cell line, Ad-mediated expression of FAST caused a modest decrease in metabolic activity in SNB75, but not 786-O (S1 Fig). In contrast, at the highest MOI of 100, treatment of the normal lung cell lines with AdFAST caused a significant decrease in metabolic activity relative to AdEmpty. These results suggest that (1) FAST protein may only be “active” in certain tumor cell types and (2) FAST protein expression should be confined only to cancer cells, as high-level expression of FAST protein in normal cells can be deleterious.

FAST protein-mediated syncytium formation is reported to cause caspase-dependent cell death with a concomitant loss in membrane integrity [14]. To determine whether apoptosis was also observed in AdFAST-treated A549 cells, we assayed for cleaved caspase 3, a marker of apoptosis, in crude extracts from treated cells. As expected, treatment of A549 cells for 24 h with 100 μM etoposide or for 6 h with 1 μM staurosporine, known inducers of caspase-mediated cell death, caused cleavage of caspase 3 (Fig 4C). However, the amount of cleaved caspase 3 was modest, and did not increase with higher doses of drug or increased incubation time (data not shown), suggesting our A549 cells are relatively resistant to these apoptotic stimuli. Treatment of cells with AdEmpty at an MOI of 10 caused evidence of caspase cleavage at both 48 and 72 hpi. However, treatment of A549 cells with AdFAST at the same MOI caused a significant increase in the amount of cleaved caspase 3 at the 72 h time point (Fig 4C). To determine if treatment of A549 cells with AdFAST also resulted in enhanced membrane permeability, we examined LDH release into the supernatant (Fig 4D). Treatment of cells with AdEmpty at an MOI up to 100 did not result in any significant release of LDH while treatment of A549 cells with AdFAST at as low an MOI as 5 led to extensive cell membrane permeability and release of LDH, which was further enhanced with increasing MOI. Thus, the reduced metabolic activity observed for AdFAST-treated cells is accompanied by onset of apoptosis and a concomitant loss in membrane integrity.

### FAST protein expression from a replication defective Ad does not improve survival in an A549 xenograft cancer model

**Fig 5:**
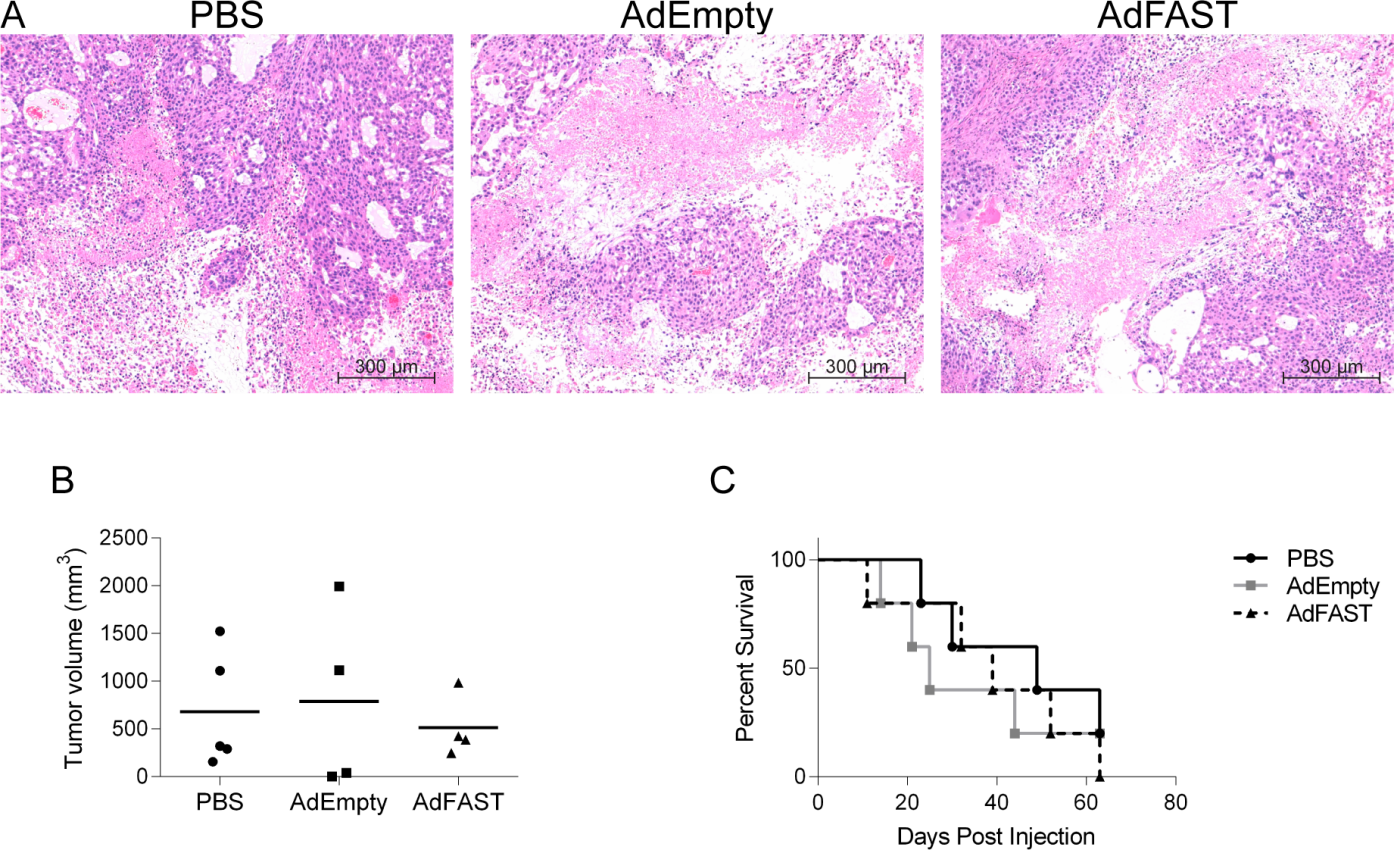
AdFAST does not induce fusion or promote survival in immunodeficient CD-1 mice with subcutaneous A549 tumors. **A)** CD-1 nude mice harbouring subcutaneous A549 tumors were intratumorally injected with PBS, 5x10^8^ pfu AdEmpty or AdFAST. Five days post injection, tumors were excised, fixed, sectioned and subjected to hematoxylin and eosin staining. Results are representative of 2–3 mice (10x objective). **B)** CD-1 mice with subcutaneous A549 tumors intratumorally injected with PBS, 5x10^8^ pfu AdEmpty or AdFAST were measured at day 20 post injection. The line in each column represents the average of the associated treatment group. **C)** A Kaplan-Meier survival curve shows the percentage survival of CD-1 mice with subcutaneous A549 tumors intratumorally injected with PBS, AdEmpty or AdFAST over time. Each treatment group consisted of 5 mice.

Since we had observed significant fusion and cell death in A549 cells infected with AdFAST *in vitro*, we examined whether FAST protein expression would be beneficial in an *in vivo* setting. CD-1 nude mice were injected with A549 cells in the right hind flank to establish subcutaneous tumors and were subsequently treated with intratumoral injections of PBS or virus. To determine whether AdFAST induced cell fusion in the A549 subcutaneous tumors, we isolated tumors from mice intratumorally injected with PBS, AdEmpty or AdFAST 5 days post injection. H/E stains showed no obvious difference in virus-injected tumors (Fig 5A). As Ad normally localizes to the liver, liver samples were also subjected to H/E staining, and no differences in pathology was observed between the various treatments (not shown). At day 20 post injection, while AdFAST injected tumors appeared to be slightly smaller in size compared to AdEmpty and PBS injected tumors, this did not reach statistical significance (Fig 5B). Furthermore, AdFAST did not improve survival of treated mice (Fig 5C). Thus, our *in vivo* studies suggest that expression of FAST protein from an E1-deleted, replication defective Ad vector is not sufficient to achieve a beneficial therapeutic effect in the A549 xenograft tumor model in immunocompromised mice.

## Discussion

Ad has shown great promise as a delivery vehicle in many animal models of human disease. Unfortunately, when injected into a solid tumor, Ad remains close to the injection tract, rarely infecting cells more than about 5 mm from the injection site, regardless of dose [2]. To overcome this problem, we investigated whether the cell-cell fusion abilities of the p14 FAST protein could enhance the cell area impacted by Ad. Cell fusion would theoretically allow spread of Ad encoded therapeutic gene products, or Ad itself, to neighbouring cells, potentially enhancing its therapeutic benefits. In addition, since fusogenic proteins can act as effective sole anti-cancer therapeutics [4,5], we also examined the effect of Ad-mediated FAST protein expression on A549 adenocarcinoma cell line viability and in a xenograft nude mouse tumor model.

After confirming FAST protein expression from our Ad constructs (Fig 1B), we examined the effects of FAST protein in 293 cells under conditions where the E1-deleted vector would replicate and yield very high levels of p14 protein. FAST protein expression caused extensive cell fusion (Fig 2) and reduced metabolic activity within the cells (Fig 2D). The exact mechanism by which FAST protein mediates cell fusion has yet to be elucidated. FAST protein traffics through the endoplasmic reticulum/Golgi complex, an event that appears necessary for homomultimerization of the protein [31]. After reaching the plasma membrane, FAST protein localizes to adherens junctions and utilizes cellular cadherins and actin remodeling to promote membrane apposition, as the very short extracellular domain of FAST does not appear to play a role in mediating pre-fusion attachment [32]. Through an uncharacterized mechanism, FAST protein promotes hemifusion, initial pore formation and expansion of these structures into stable micropores, and finally syncitium formation. A subfragment of the FAST protein, the 68-residue endodomain generated naturally from proteolytic processing of the full-length protein, can function to enhance pore expansion [33], a process that is sensitive to the membrane curvature-inducing lipid lysophosphatidylcholine [34], and that requires cellular annexin A1 [35]. FAST protein-mediated fusion is reported to be a relatively non-leaky process (i.e. the cell membrane remains largely intact until late in the process); however, extensive syncytium formation induces cell death, which itself may be responsible for the altered membrane integrity [14]. For the GALV fusion protein, syncytia remain viable for up to 2 days after fusion, but then suffer from a collapse of mitochondrial membrane potential and a concomitant severe depletion of ATP which leads to cell death [36]. It was speculated that the GALV may be in part mislocalized to the mitochondria, affecting mitochodrial membrane integrity and ultimately leading to the observed depolarization. Whether FAST protein also impacts upon organelle function, prior to the onset of cell death, has not yet been investigated.

The extensive cell fusion caused by high-level expression of the FAST protein from a replicating Ad vector suggests that the FAST protein may be appropriate for inclusion in oncolytic Ad vectors. In that context, FAST protein expression may help promote spread of the virus throughout the tumor mass, thus circumventing one of the drawbacks of Ad and many other vector systems. For example, complete tumor regression can be achieved with the oncolytic adenovirus ONYX-015 with initial infection of as little as 5% of the tumor cells, but these cells must be equally distributed throughout the tumor [37]. Consequently, distribution and efficacy can be improved through use of multiple, sequential injections of vector [37]. FAST protein expression from an oncolytic Ad would not only allow the virus to spread through normal routes (i.e. tumor cell lysis and virus release), but an infected cell can “spread” its infectious payload through direct cell-cell fusion. The small size of the FAST cDNA of only 375 bp, means that large cancer-relevant regulator regions, such as the telomerase reverse transcriptase (TERT) promoter [38], could be easily accommodated in the vector for tumor-specific regulation of the essential E1 region. Alternatively, immunostimulatory genes could be accommodated in an AdFAST oncolytic vector to enhance the therapeutic effect. Such a virus would function through multiple modalities to achieve tumor cell killing and would potentially be more effective than current oncolytic Ad.

In A549 cells, which are non-permissive for replication of these E1-deleted vectors, FAST protein expression caused cell-cell fusion only at relatively high MOI (Fig 3), suggesting that there may be a threshold of FAST expression required for fusion. FAST-mediated fusion requires oligomerization of the protein through its ectodomain, which could explain why high level FAST protein expression is required for its function [31]. The idea of a threshold may be beneficial in the context of an oncolytic Ad where selective replication will increase FAST protein expression to levels high enough to induce fusion only in cancer cells, whereas low level, spurious expression in normal cells would be expected to have little effect. Consistent with this, we observed that a high MOI of AdFAST was required to affect metabolic activity in normal human cells, while low MOI had no effect (S1 Fig). Thus, regulated expression of FAST protein from an oncolytic vector may provide an effective means to enhance Ad distribution through the tumor mass, in addition to contributing to tumor cell killing directly.

In A549 cells, a significant decrease in metabolic activity was observed in AdFAST- versus AdEmpty-treated cells (Fig 4A and 4B), and the cells underwent apoptosis (Fig 4C) with a concomitant loss of membrane integrity (Fig 4D). Interestingly, compromised membrane integrity was noted at an MOI of 5 or greater, conditions that did not induce extensive syncytia formation. In a study by Salsman *et al*. [14], overexpression of FAST proteins, including the reptilian reovirus p14 protein, Avian reovirus p10 protein and Nelson Bay reovirus p10 protein in Vero and QM5 cells, caused decreased cell membrane integrity only after cell fusion and initiation of apoptosis. Moreover, membrane integrity could be rescued through use of an apoptotic inhibitor, suggesting decreased membrane integrity was a result of cell death rather than of FAST protein expression. However, studies of the Avian reovirus p10 protein in monkey BSC-40 cells showed that expression of a truncated version of p10 FAST protein compromised membrane integrity but did not induce cell-cell fusion [39]. These data suggest that effects on membrane leakiness and cell fusion may be two independent events. Differences in cell lines and expression conditions may cause the FAST proteins to induce membrane permeabilization without extensive cell-cell fusion, as seen in our study. However, in our experiments, it is also possible that FAST protein expression caused formation of small syncytia at low MOIs, which were not detected in our fusion assays, leading to the membrane permeability detected. Nevertheless, FAST protein induced membrane permeability suggests that non-replicating AdFAST may be useful when used in combination with chemotherapeutic drugs, to enhance their entry into cells, thereby achieving a greater therapeutic benefit at a reduced drug dosage.

In our nude mouse studies, we did not see any obvious signs of fusion in AdFAST injected tumors (Fig 5A). Furthermore, treatment with AdFAST did not lead to a significant decrease in tumor burden or enhance survival compared to control mice (Fig 5B and 5C). Our *in vitro* studies suggested that high level FAST protein expression is required to achieve significant fusion in A549 cells (Fig 3). It is possible that the difference between the tumor microenvironment and *in vitro* conditions affects gene expression from the Ad vector, resulting in lower relative expression of FAST protein *in vivo* compared to *in vitro*. Alternatively, the effective multiplicity of infection for cells contained in a tumor, and hence the level of FAST protein expression, may not reach the threshold level required for efficient fusion. Under conditions of active viral replication, FAST protein expression is higher (Fig 1B) and associated with enhanced fusion (Fig 2) and decreased metabolic activity (Fig 2D). This observation once again suggests that FAST protein expression may provide a better effect in the context of a conditional-replicating, oncolytic Ad vector. It is also important to note that, in this study, we are utilizing a tumor model based on immunodeficient mice. Previous studies have shown that fused human cancer cells and the exosome-like syncytiosomes that they release can promote dendritic cell cross presentation and antigen specific T-cell activation [4,40]. Thus, AdFAST may provide a greater therapeutic benefit in an immunocompetent cancer model, where FAST protein-mediated cell fusion and subsequent syncytiosome release may aid in stimulating anti-tumor immunity.

In this study, we showed that Ad vectors can be used to express the p14 FAST fusogenic protein in cancer cells, resulting in direct cell-cell fusion, compromised cell membrane integrity, and enhanced cell death. Our data shows that the ability of the FAST protein to fuse cells, compromise membrane integrity and promote apoptosis is directly related to the FAST protein expression level and cell type. Although a replication-defective vector expressing the FAST protein did not provide a significant therapeutic benefit in a xenograft model of cancer, our study suggests that the FAST protein may enhance the efficacy and spread of oncolytic Ad, where vector replication will increase FAST protein expression, or synergize with chemotherapeutic drugs to enhance their uptake and cancer cell killing.

## Supporting Information

S1 Fig Effect of AdFAST on metabolic activity in a variety of human cell lines. **A)** 786-O and SNB75 cells were infected at varying MOIs with AdEmpty or AdFAST and the relative metabolic activity was determined at 72 hpi. Three independent experiments were conducted in triplicate and the average is shown with the standard error of the mean (n = 3). Experiments were conducted in the same way as described in the MTS metabolic activity assays section of the Materials and Methods using DMEM media. *p<0.05 compared to PBS-treated cells. **p<0.05 comparing AdFAST to AdEmpty treated cells. **B)** Patient-derived normal primary lung cell lines U97N and U116N were subjected to the same experiment as described in Panel A. These cells were obtained at passage 2–4, and used for these experiments at less than passage 10. Results show the average of three replicates with the standard error of the mean. *p<0.05 compared to PBS-treated cells. **p<0.05 comparing AdFAST to AdEmpty treated cells. **C and D)** To confirm FAST protein expression, cells were infected with AdEmpty or AdFAST-HA at an MOI or 100 (or mock infected with PBS) and crude protein extracts were collected 72 hr later and assayed for FAST expression by immunoblot for the HA tag. As a loading control, the membranes were also probed with antibody to β-actin.
(TIF)

S1 Movie Live-imaging analysis of 293 cells infected with AdRFP. 293 cells were infected at an MOI of 1 with AdRFP and subjected to live-imaging analysis 12 to 46 hpi using the Zeiss Axiovert 200M microscope with a 20x objective in a 37°C chamber with 5% CO2.
(MOV)

S2 Movie Live-imaging analysis of 293 cells infected with AdFAST. 293 cells were infected at an MOI of 1 with AdFAST/RFP. Live imaging was conducted in a 37°C chamber supplemented with 5% CO2. Images were taken from 12 hpi to 46 hpi at half hour intervals using the Zeiss Axiovert 200M microscope with a 20x objective.
(MOV)
