# Supplementary figures and images for: Adenovirus-Mediated Expression of the p14 Fusion-Associated Small Transmembrane Protein Promotes Cancer Cell Fusion and Apoptosis In Vitro but Does Not Provide Therapeutic Efficacy in a Xenograft Mouse Model of Cancer
Source: PLoS One. 2016 Mar 17;11(3):e0151516. doi: 10.1371/journal.pone.0151516 (PMC4795661; doi:10.1371/journal.pone.0151516)

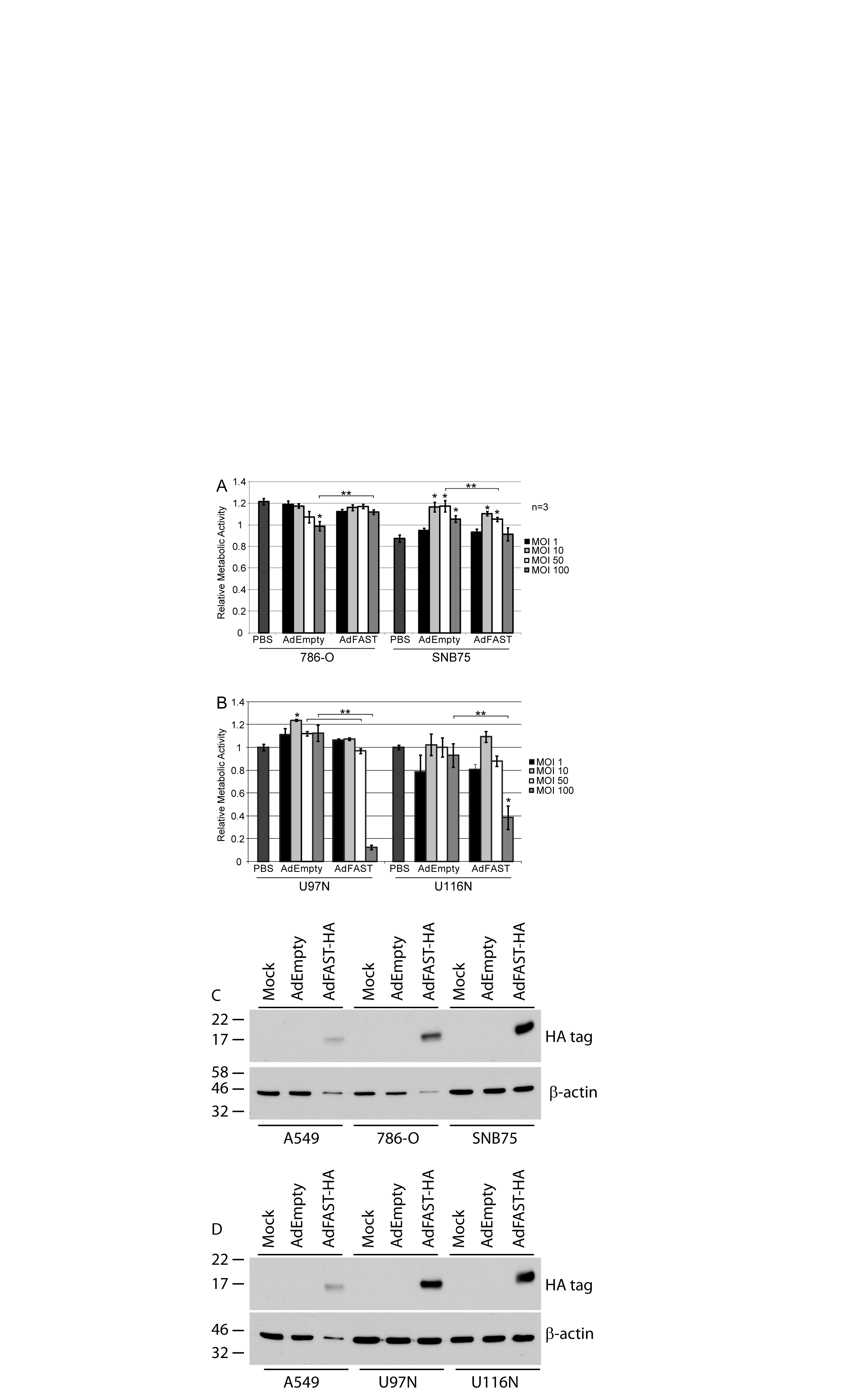

Supplement: S1 Fig — A) 786-O and SNB75 cells were infected at varying MOIs with AdEmpty or AdFAST and the relative metabolic activity was determined at 72 hpi. Three independent experiments were conducted in triplicate and the average is shown with the standard error of the mean (n = 3). Experiments were conducted in the same way as described in the MTS metabolic activity assays section of the Materials and Methods using DMEM media. *p<0.05 compared to PBS-treated cells. **p<0.05 comparing AdFAST to AdEmpty treated cells. B) Patient-derived normal primary lung cell lines U97N and U116N were subjected to the same experiment as described in Panel A. These cells were obtained at passage 2–4, and used for these experiments at less than passage 10. Results show the average of three replicates with the standard error of the mean. *p<0.05 compared to PBS-treated cells. **p<0.05 comparing AdFAST to AdEmpty treated cells. C and D) To confirm FAST protein expression, cells were infected with AdEmpty or AdFAST-HA at an MOI or 100 (or mock infected with PBS) and crude protein extracts were collected 72 hr later and assayed for FAST expression by immunoblot for the HA tag. As a loading control, the membranes were also probed with antibody to β-actin. (TIF) [file pone.0151516.s001.tif]
